# Shp2 regulates migratory behavior and response to EGFR-TKIs through ERK1/2 pathway activation in non-small cell lung cancer cells

**DOI:** 10.18632/oncotarget.20249

**Published:** 2017-08-14

**Authors:** Yu-Jing Sun, Zhong-Ling Zhuo, Hai-Peng Xian, Ke-Zhong Chen, Fan Yang, Xiao-Tao Zhao

**Affiliations:** ^1^ Department of Clinical Laboratory, Peking University People’s Hospital, Beijing 100044, China; ^2^ Department of Clinical Laboratory, Peking University International Hospital, Beijing 100044, China; ^3^ Department of Thoracic Surgery, Peking University People’s Hospital, Beijing 100044, China

**Keywords:** Shp2, non-small cell lung cancer, epidermal growth factor receptor, tyrosine kinase inhibitors, ERK1/2

## Abstract

In the clinical treatment of lung cancer, therapy failure is mainly caused by cancer metastasis and drug resistance. Here, we investigated whether the tyrosine phosphatase Shp2 is involved in the development of metastasis and drug resistance in non-small cell lung cancer (NSCLC). Shp2 was overexpressed in a subset of lung cancer tissues, and Shp2 knockdown in lung cancer cells inhibited cell proliferation and migration, downregulated c-Myc and fibronectin expression, and upregulated E-cadherin expression. In H1975 cells, which carry double mutations (L858R + T790M) in epidermal growth factor receptor (EGFR) that confers resistance toward the tyrosine kinase inhibitor gefitinib, Shp2 knockdown increased cellular sensitivity to gefitinib; conversely, in H292 cells, which express wild-type EGFR and are sensitive to gefitinib, Shp2 overexpression increased cellular resistance to gefitinib. Moreover, by overexpressing Shp2 or using U0126, a small-molecule inhibitor of extracellular signal-regulated kinase 1/2 (ERK1/2), we demonstrated that Shp2 inhibited E-cadherin expression and enhanced the expression of fibronectin and c-Myc through activation of the ERK1/2 pathway. Our findings reveal that Shp2 is overexpressed in clinical samples of NSCLC and that Shp2 knockdown reduces the proliferation and migration of lung cancer cells, and further suggest that co-inhibition of EGFR and Shp2 is an effective approach for overcoming EGFR T790M mutation acquired resistance to EGFR tyrosine kinase inhibitors (TKIs). Thus, we propose that Shp2 could serve as a new biomarker in the treatment of NSCLC.

## INTRODUCTION

Lung cancer is a leading cause of cancer death worldwide. Lung cancer has been reported to account for 13% of all new tumor cases [1], and 50% of patients with stage I or II non-small cell lung cancer (NSCLC) have been found to develop systemic metastases despite complete resection [2]. Traditional chemotherapy is only modestly effective in the treatment of patients with advanced lung cancer, with the median survival time being only 8–10 months [3]. Recently, targeted therapies involving the use of epidermal growth factor receptor-tyrosine kinase inhibitors (EGFR-TKIs) were reported to be beneficial for NSCLC patients, but the response to EGFR-TKIs was limited mainly to NSCLC patients carrying EGFR mutations (50% of Asian patients and 15% of Western patients), and ∼20%–30% of these patients failed to respond to the drugs [4]. Furthermore, some of these patients might develop resistance to EGFR-TKIs, frequently through the EGFR T790M mutation or through upregulation of c-MET or other receptors [5]. Consequently, only 10%–20% of NSCLC patients might benefit from EGFR-TKIs treatments, and improved therapeutic approaches are clearly required. Thus, to enable the development of new and effective targeted therapeutic drugs for lung cancer, considerable research effort is currently being devoted toward enhancing our understanding of the mechanisms and molecules that regulate the growth and migratory behavior of lung cancer cells.

Protein tyrosine phosphorylation and dephosphorylation levels in cells are governed by the balanced actions of protein tyrosine kinases (PTKs) and protein tyrosine phosphatases (PTPs) [6]. Whereas PTKs have been widely reported to promote the development of human cancers, PTPs have been mostly regarded as tumor suppressors. However, increasing evidence suggests that certain PTPs can also promote cancer development (i.e., act as oncogenes), such as the PTP Shp2 (src homology 2 domain-containing tyrosine phosphatase 2), which is encoded by *PTPN11* [7]. Shp2—the first PTP-superfamily member identified to act as an oncogene—functions in the control of cell proliferation, survival, differentiation, invasion, metastasis, and morphogenesis [8].

Shp2 is a ubiquitously expressed PTP that participates in signaling events proximal to receptor PTKs, such as EGFR and PDGF, insulin, and IGFI hematopoietic receptors [9]. Activated Shp2 has been reported to mediate growth factor-stimulated Ras-ERK1/2 (extracellular signal-regulated kinase 1/2) activation and promote cell growth and survival [7]. How Shp2 mediates Ras-ERK1/2 activation remains incompletely understood, although the activation could involve several potential mechanisms, including the dephosphorylation of a RASGAP binding site on GAB1 or the dephosphorylation of CSK binding sites on PAG/CBP and paxillin [10, 11]. Because Shp2 functions in multiple oncogenic receptor PTK pathways, targeting Shp2 could represent a favorable strategy for improving cancer therapies.

Shp2 has now been widely confirmed to represent a promising target in cancer treatment [12–15], and Shp2 has been recently suggested to function in tumor initiation and to enhance tumor maintenance and progression [16–19]. However, no previous study has comprehensively evaluated Shp2 function in NSCLC development. We hypothesized that Shp2 expression contributes to NSCLC progression and that Shp2 targeting could serve as a potential treatment for lung cancer. In previous work, we have focused on PTP actions in diverse signaling pathways and diseases [20–22]. Here, we specifically investigated the function of Shp2 in lung cancer by manipulating Shp2 expression in lung cancer cell lines.

## RESULTS

### Shp2 is overexpressed in clinical samples of NSCLC

**Figure 1:**
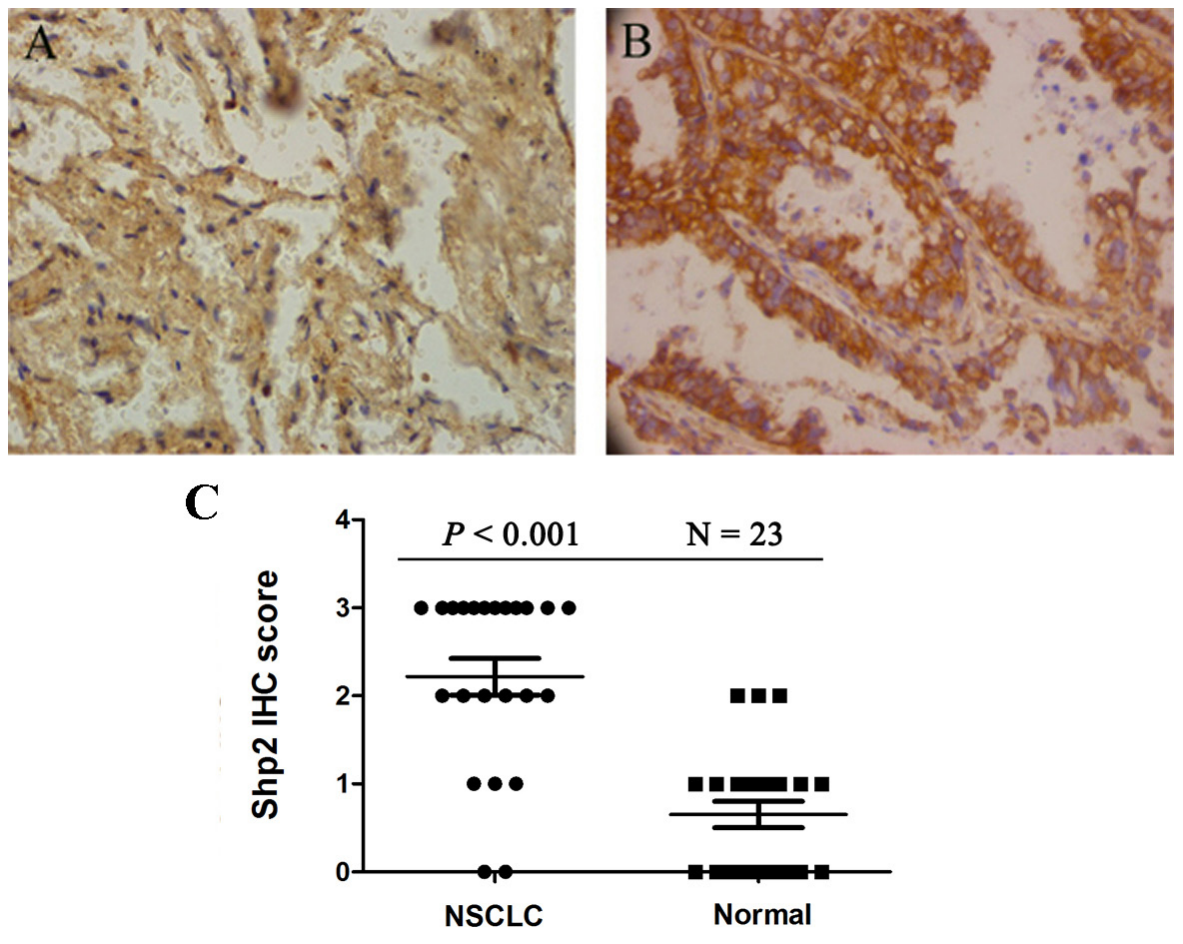
Shp2 expression is increased in non-small cell lung cancer (NSCLC) Anti-Shp2 antibody staining of **(A)** normal lung tissue and **(B)** NSCLC tissue. The IHC semi-quantitative score was derived based on two criteria: the antibody staining intensity was multiplied by the percentage of tumor cells stained. IHC scores for each set of specimens were averaged (N = 23) and statistically analyzed **(C)**.

To assess the potential involvement of Shp2 in NSCLC development, we first examined Shp2 expression in human NSCLC tumor tissues and paired adjacent normal tissues. Immunohistochemical staining of 23 distinct tissue samples revealed that Shp2 expression was significantly higher in lung cancer tissues than in normal lung tissues (*P* < 0.001) (Figure 1).

### Shp2 knockdown inhibits tumor growth and enhances cellular response to gefitinib

**Figure 2:**
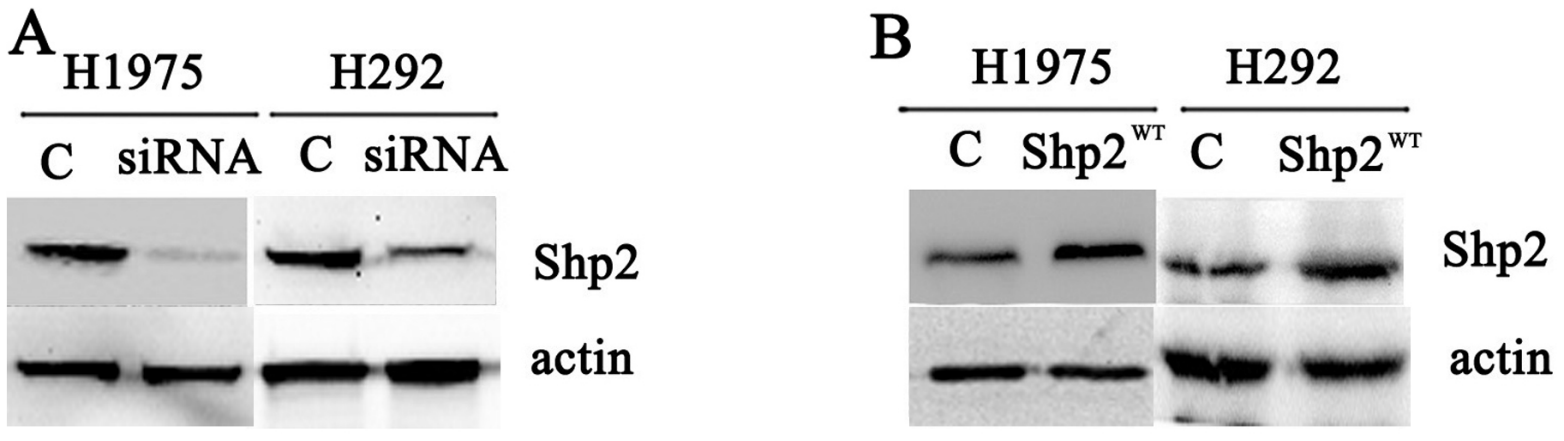
Transfection of Shp2 siRNA and Shp2^WT^ cDNA decreased and increased cellular Shp2 expression, respectively Equal amounts of lysates prepared from cells transfected with control/Shp2 siRNAs **(A)** or vector/Shp2^WT^ **(B)** were immunoblotted with anti-Shp2 and anti-actin antibodies (N = 3).

**Figure 3:**
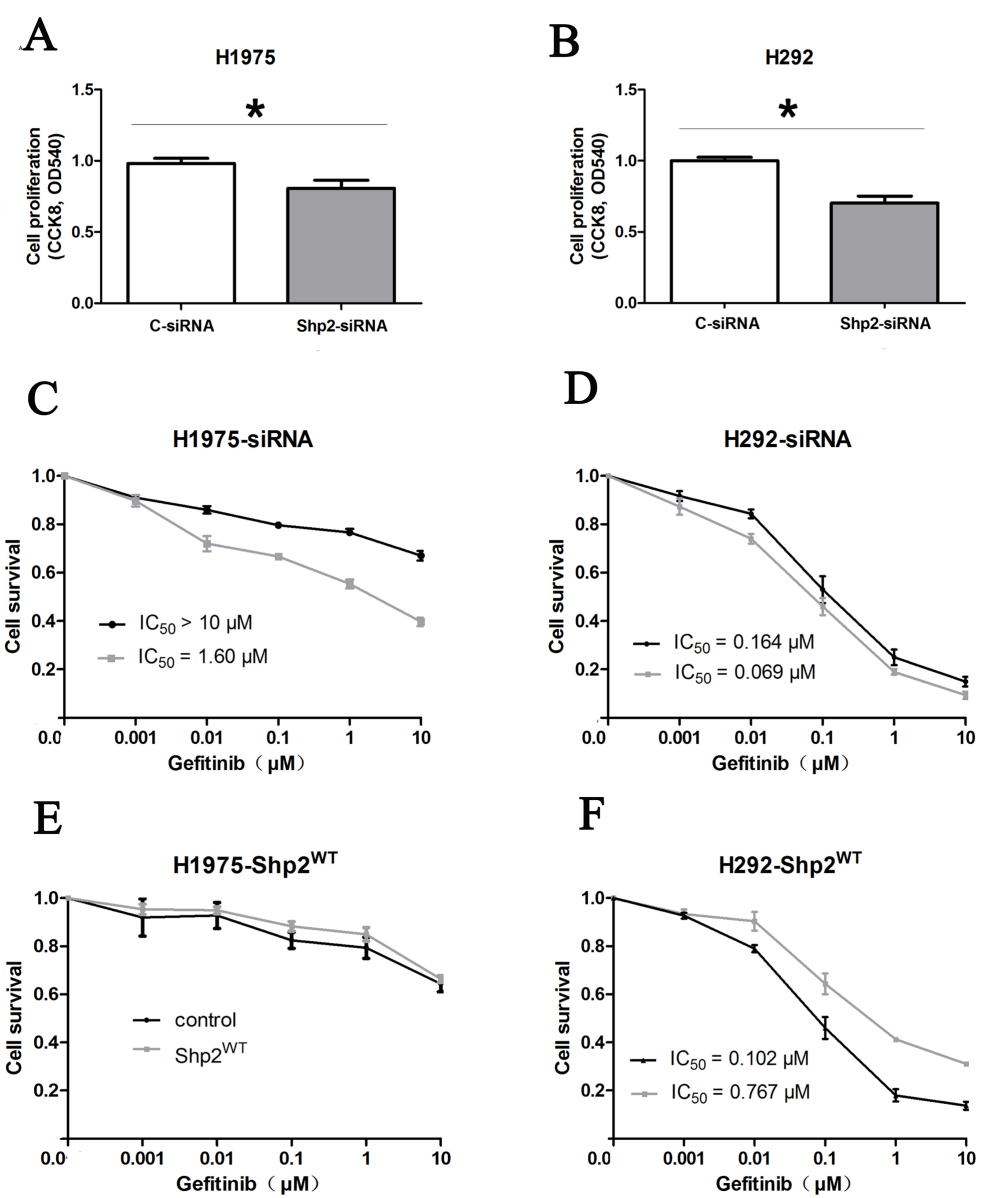
Shp2 knockdown inhibits tumor growth and enhances cellular response to gefitinib **(A)**, **(B)** Shp2 knockdown decreased the proliferation of H1975 cells (A) and H292 cells (B). Lung cancer cells were transfected with Shp2 or control siRNA for 72 h in RPMI 1640 medium containing 0.5% FBS. Cell proliferation was measured using the CCK8 assay at 72 h after transfection, and the results are expressed as means ± SD (N = 4); *P < 0.05 versus control. **(C)**, **(D)** Shp2 knockdown enhanced gefitinib sensitivity in NSCLC cells. **(E)**, **(F)** Shp2 overexpression decreased gefitinib sensitivity in NSCLC cells. H1975 and H292 cells transfected with Shp2/control siRNAs or Shp2^WT^/vector were treated with 0–10 μM gefitinib for 3 days, and then cell proliferation was measured using the CCK8 assay. The cell-viability percentage was calculated by adjusting the DMSO control group to 100%. Points and error bars represent the mean and SD values from 3 independent experiments. Black points: control siRNAs/vector; Gray points: Shp2-siRNAs/Shp2^WT^.

We examined the functional significance of Shp2 expression in NSCLC cells by knocking down Shp2 expression through RNA interference in the cell lines H1975 and H292 (Figure 2A). In H1975 cells, which are gefitinib resistant, proliferation was decreased following Shp2 siRNA transfection, and the IC50 of the cellular response to gefitinib was reduced from >10 μM to 1.60 μM after Shp2 depletion (Figure 3A, 3C). Cell proliferation was also markedly inhibited in the case of Shp2-depleted H292 cells, but these cells were only slightly sensitized to gefitinib after Shp2 knockdown (Figure 3B, 3D). In a complementary set of assays, we examined the effect of Shp2 overexpression in cells (Figure 2B); whereas expression of Shp2^WT^ decreased cellular sensitivity to gefitinib in H292 cells (Figure 3F), the expression did not alter gefitinib sensitivity in H1975 cells (Figure 3E).

### Shp2 knockdown reduces migration in NSCLC cells

**Figure 4:**
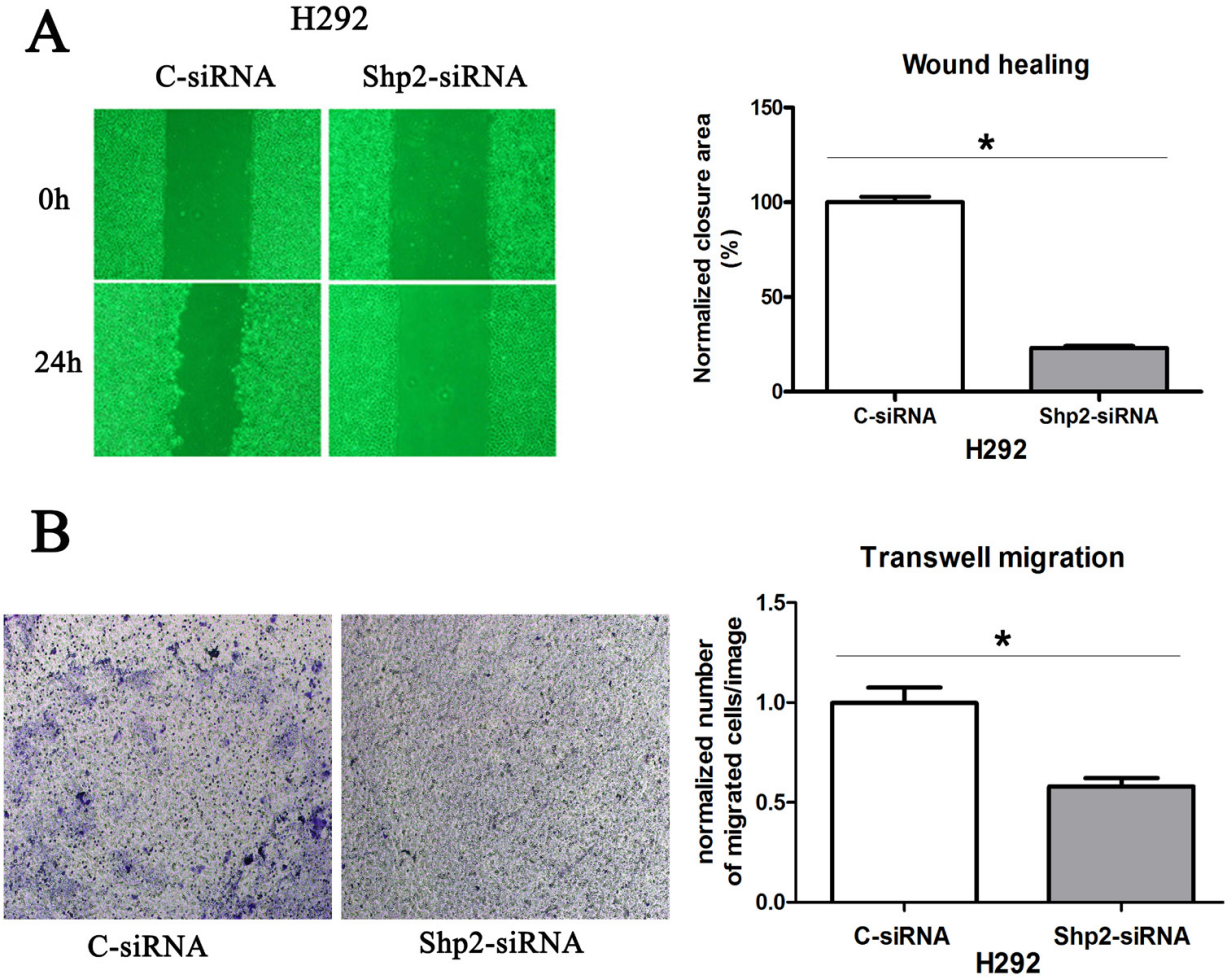
Shp2 knockdown inhibits the migration of lung cancer cells Cells transfected with Shp2/control siRNAs were used in wound-healing assays **(A)** and Transwell-migration assays **(B)**. In the wound-healing assay, cell images were captured immediately after wounding and 24 h later. In Transwell-migration assays, equal numbers cells were cultured on Transwell membranes for 24 h, and then the migrated cells were counted. *P < 0.05 versus controls.

Next, Shp2 involvement in the control of NSCLC cell migration was assessed by manipulating Shp2 expression and performing wound-healing and Transwell-migration assays. When Shp2 expression was downregulated in H292 cells (Figure 2A), wound closure was slowed (Figure 4A) and migration through the Transwell membrane was delayed (Figure 4B). However, Shp2^WT^ expression in H292 cells caused no increase in cell migration in either the wound-healing assay (data not shown) or the Transwell-migration assay (data not shown), which suggests that the parental cancer cell line already exhibits maximal migration capacity.

### Shp2 expression enhances c-Myc expression

**Figure 5:**
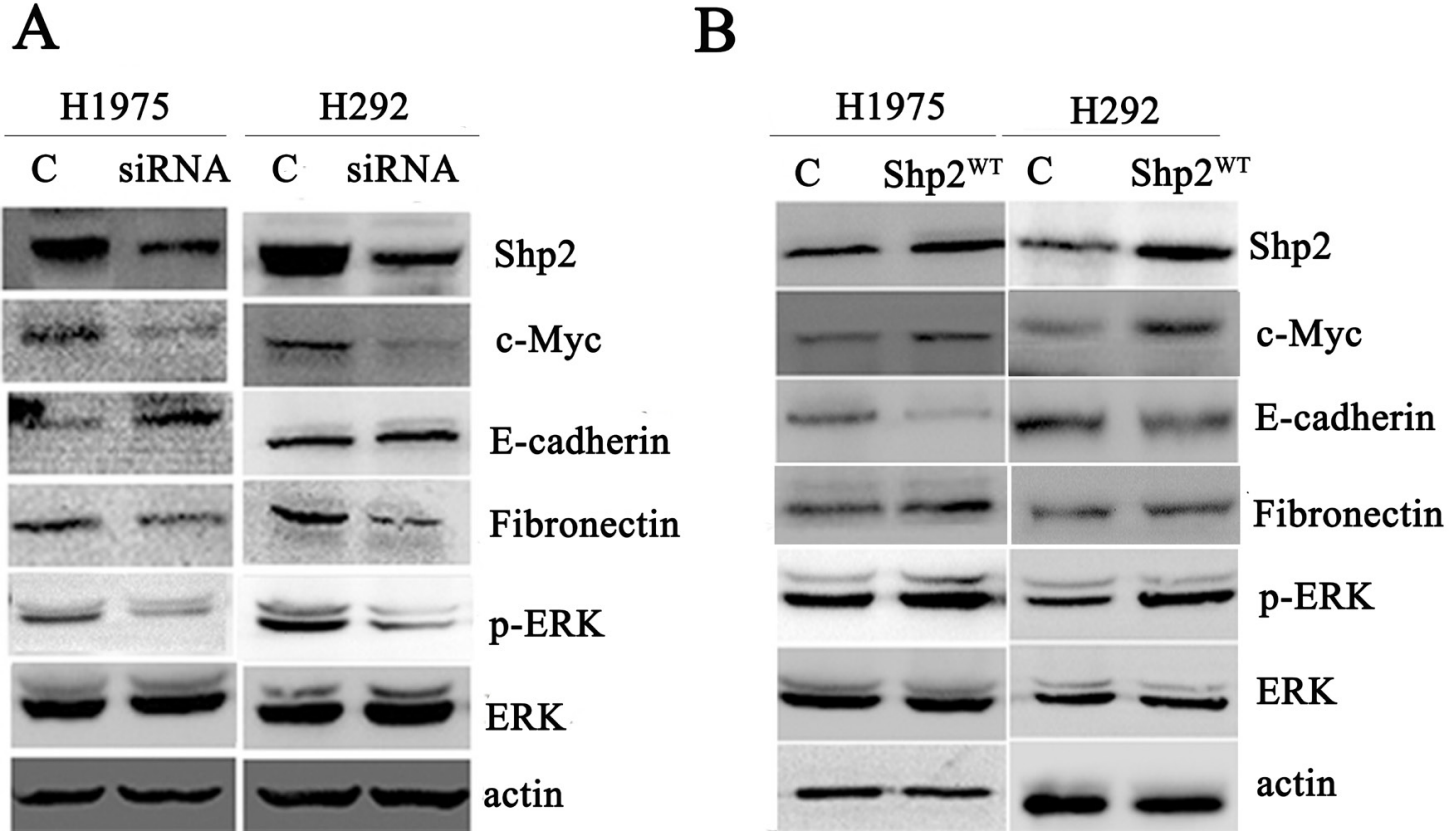
Shp2 expression enhances c-Myc expression and EMT Shp2 siRNA transfection reduced c-Myc and fibronectin expression, enhanced E-cadherin expression, and decreased ERK1/2 activation **(A)**. Shp2 overexpression reduced E-cadherin expression, enhanced fibronectin and c-Myc expression, and induced ERK1/2 activation **(B)**. Equal amounts of lysates from cells transfected with control/Shp2 siRNAs or vector/Shp2^WT^ were immunoblotted with the indicated antibodies (N = 3).

The c-Myc oncogene is frequently overexpressed in several types of cancer, and c-Myc overexpression is associated with poor prognosis in lung adenocarcinoma [23]. We performed immunoblotting to examine c-Myc expression in lung adenocarcinoma cells transfected with Shp2/control siRNA or Shp2^WT^/empty vector (Figure 5A, 5B). Whereas Shp2 knockdown resulted in c-Myc downregulation, Shp2 overexpression led to increased c-Myc expression (relative to control siRNA or vector transfection, respectively). Thus, Shp2 expression promoted c-Myc expression in lung adenocarcinoma cells.

### Shp2 promotes epithelial-to-mesenchymal transition (EMT) and Shp2 inhibition suppresses EMT

Based on considering the aforementioned results, we evaluated whether Shp2 affects the expression of the EMT-associated proteins E-cadherin and fibronectin in lung cancer cells. Following transfection of the Shp2-silencing siRNA, E-cadherin expression was increased in H1975 cells and fibronectin expression was decreased in H292 cells (Figure 5A). By contrast, E-cadherin expression was decreased in cells expressing Shp2^WT^ (Figure 5B). These results indicate that Shp2 promotes EMT and that Shp2 inhibition leads to mesenchymal-to-epithelial transition in lung cancer cells.

### Shp2 enhances c-Myc expression and EMT potentially through Ras/MAPK signaling

To identify the potential signaling pathways by which Shp2 upregulates c-Myc expression and promotes EMT, we examined the activation of the Ras/MAPK pathway, one of the key signaling pathways stimulated by Shp2 in other cell types. Ras/MAPK pathway activation was assessed by monitoring the phosphorylation (and thus the activation) status of the main downstream effector ERK1/2. Whereas ERK1/2 phosphorylation was higher in lung adenocarcinoma cells overexpressing Shp2 than in vector-control cells, ERK1/2 phosphorylation was lower in cells transfected with the Shp2 siRNA than in cell transfected with the control siRNA (Figure 5).

**Figure 6:**
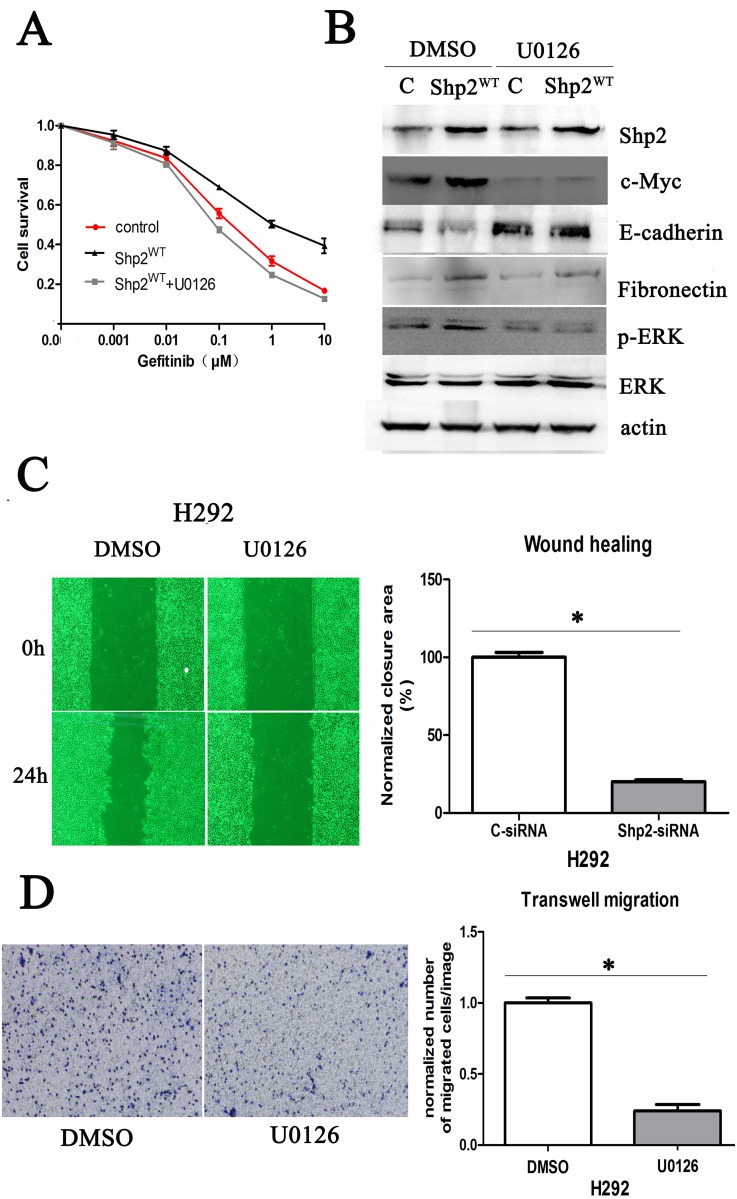
Shp2 promotes c-Myc expression and EMT through Ras/MAPK signaling **(A)** Comparison of gefitinib sensitivity of H292 cells transfected with vector or Shp2^WT^ and treated with DMSO or U0126; cell survival was measured using the CCK8 assay. **(B)** U0126 treatment abrogated Shp2^WT^-induced upregulation of c-Myc and fibronectin expression and downregulation of E-cadherin expression in H292 cells. Equal amounts of lysates from cells transfected with vector/Shp2^WT^ were immunoblotted with the indicated antibodies. **(C)**, **(D)** U0126 inhibits H292 cell migration; cells treated with DMSO or U0126 were used in wound-healing assays (C) and Transwell-migration assays (D). *P < 0.05 versus controls.

Lastly, to determine whether Shp2 promotes c-Myc expression and EMT through activation of ERK1/2 signaling, we tested the effect of U0126, a small-molecule inhibitor of ERK1/2 (Figure 6). Western blotting analysis revealed that Shp2^WT^-induced upregulation of c-Myc and fibronectin expression and downregulation of E-cadherin expression were abrogated in H292 cells treated with U0126 (Figure 6B). Accordingly, in H292 cells expressing Shp2^WT^, the decreased sensitivity to gefitinib caused by Shp2 overexpression was counteracted by U0126 (Figure 6A). Moreover, we tested whether U0126 can block the migration of NSCLC cells. Because Shp2 overexpression did not markedly affect cell migration in this study (as noted earlier in this section), we used the parental H292 cells, and we quantified the motility of the cells in the absence or presence of U0126 by using wound-healing and Transwell-migration assays. Our results showed that U0126 was as potent as the Shp2 siRNA in suppressing the migration and invasive behavior of H292 cells (Figure 6C, 6D).

## DISCUSSION

We have demonstrated here that Shp2 was overexpressed in lung cancer tissue samples from a group of NSCLC patients from Beijing, and that in lung cancer cells, Shp2 promoted proliferation, reduced gefitinib sensitivity, and enhanced migration through activation of the ERK1/2 pathway. These results suggest that Shp2 functions as a key positive regulator of lung cancer progression. More than 58 different Shp2 mutations have been identified in various tumors, and in patients with these tumors, normal cell proliferation and migration are disrupted [8]. However, in lung cancer, the Shp2 mutation rate is only 1.81% according to the Catalogue of Somatic Mutations in Cancer databank (www.sanger.ac.uk) [24]. Therefore, our finding here that wild-type Shp2 overexpression enhances tumor growth and migration reveals a broad role of Shp2 in promoting the progression of lung cancer.

The c-Myc oncogene is regarded as a driver oncogene that links growth factor stimulation to proliferation in normal and tumor cells [25]. Our results showed that Shp2 knockdown inhibited c-Myc expression, and, accordingly, proliferation was impaired in Shp2-knockdown cells. Moreover, c-Myc is frequently overexpressed in lung cancer and promotes tumor progression in Raf- or Ras-driven lung cancer, and this is associated with poor prognosis [26, 27]. Our results also indicated that ERK1/2 signaling, which occurs downstream of Shp2 and Ras, acted upstream of c-Myc. Thus, Shp2 might regulate lung cancer cell proliferation through the ERK/c-Myc signaling axis.

EGFR-TKIs treatment is associated with survival in NSCLC patients harboring EGFR activating mutations [28]. However, despite a highly favorable initial response, drug resistance and tumor progression occur in most patients [29, 30]. Because Shp2 functions as a mediator of EGFR signaling, we investigated whether Shp2 affects cellular sensitivity to the EGFR-TKIs gefitinib. In gefitinib-resistant H1975 cells, Shp2 knockdown markedly lowered the gefitinib IC50, this finding indicate that combined inhibition of EGFR and Shp2 might improve drug response in tumors harboring the T790M mutation. Whereas the Shp2 knockdown only slightly affected the gefitinib sensitivity of H292 cells, which are gefitinib-sensitive lung cancer cells. Given that Shp2 promotes tumor growth and migration through the activation of the ERK pathway [7], the aforementioned findings raise the intriguing question of whether EGFR-TKIs completely inhibit ERK activity. The T790M mutation causes gefitinib resistance by sterically interfering with gefitinib binding to EGFR, which can result in diminished impaired ERK phosphorylation in H1975 cells relative to the H292 cells that are sensitive to gefitinib [31]. In H1975 cells, Shp2 knockdown reduced ERK phosphorylation by >70%. This result suggests that ERK inhibition by using only EGFR-TKIs might not be effective in certain cases, and that co-inhibition of EGFR and Shp2 might represent a comparatively more effective strategy for the treatment of gefitinib-resistant lung tumors. The finding here that Shp2 overexpression mitigated the effects of gefitinib by enhancing ERK phosphorylation in H292 cells furthers bolsters our conclusion. Moreover, growing evidence indicates that EMT increases resistance to EGFR-TKIs therapy [5, 32, 33], and our results showed that Shp2 knockdown induced E-cadherin upregulation and fibronectin downregulation, which are characteristic of mesenchymal-to-epithelial transition and have been found to be associated with improved response to EGFR-TKIs treatment.

Metastasis is the main cause of death in the majority of patients with NSCLC [34]. In this study, we evaluated critical stages in the invasive metastasis cascade, including EMT. EMT has been widely demonstrated to be frequently “hijacked” during metastatic progression through the loss of epithelial cell-junction proteins, including E-cadherin, and the gain of mesenchymal markers, such as vimentin and fibronectin [15, 35]. We observed similar changes here in lung cancer cells. Notably, Shp2 knockdown suppressed the migration of lung cancer cells, which agrees with previous work indicating that positive expression of Shp2 was closely related to the metastasis of NSCLC to lymph nodes [36]. Thus, interference with Shp2 function might provide an approach for inhibiting cancer cell metastasis. However, we did not detect a major effect of Shp2 overexpression on metastasis in lung cancer cells. Shp2 was found to be expressed at higher levels in the majority of NSCLC specimens than in adjacent normal lung samples [36]. Therefore, one underlying reason for the lack of effect of Shp2 overexpression might be that the metastatic ability of the lung cancer cells was already enhanced maximally.

Our results indicated that Shp2 expression potentially induced the cellular EMT program through activation of the ERK pathway. ERK function in EMT has been examined not only in normal development, but also in cancer metastasis. For example, ERK activation promoted the initiation of epithelial tubule development through morphological changes [37], and ERK2 was specifically implicated as an EMT driver [38]. Considering that U0126 suppressed Shp2-induced EMT and migration of lung cancer cells in this study, drugs that target the ERK pathway could potentially be used for treating Shp2-overexpressing lung cancers.

Our finding that increased Shp2 expression promotes the EMT phenotype and c-Myc expression in lung cancer cells suggests that Shp2 can serve as a potential target in lung cancer treatment. We have provided evidence indicating that Shp2 knockdown reduces the proliferation and migration of lung cancer cells and that co-inhibition of EGFR and Shp2 can effectively overcome acquired EGFR-TKIs resistance. These findings provide a rationale for future investigations into the effects of small-molecule inhibitors of Shp2 on lung cancer progression and thus into a promising new target for lung cancer therapy.

## MATERIALS AND METHODS

### Tumor tissue samples

Twenty-three pairs of primary resected NSCLC tumor specimens and control normal tissue samples, which were adjacent to and at least 5 cm from the tumor lesion, were obtained after surgical resection from patients of Peking People’s University Hospital. This study was approved by the Institutional Review Board of Peking People’s University Hospital.

### Immunohistochemistry (IHC)

IHC was performed to evaluate Shp2 expression in paraffin-embedded cancer tissue specimens and adjacent normal specimens (normal controls). Sections were stained with an anti-Shp2 antibody (1:200; sc-280, Santa Cruz Biotechnology, Santa Cruz, CA). The IHC semi-quantitative score was derived based on two criteria: Immunoreactivity was classified by estimating the percentage (P) of cancer cells exhibiting characteristic staining (from 0% to 100%) and by estimating the intensity (I) of staining (1, weak; 2, moderate; 3, strong), and then the score was calculated by multiplying the percentage of positive cells by the intensity: Q = P × I (maximum = 3).

### Cell culture and reagents

The human NSCLC cell lines H292 (gefitinib-sensitive) and H1975 (gefitinib-resistant) were maintained in RPMI 1640 medium supplemented with 100 U/mL penicillin, 100 mg/mL streptomycin, and 10% fetal bovine serum (FBS; Gibco, Gaithersburg, MD) at 37°C in a 5% CO_2_ atmosphere. The ERK1/2 pharmacological inhibitor U0126 was purchased from Calbiochem (Merck, Darmstadt, Germany) and the EGFR-TKIs gefitinib (Iressa) was from Tocris Bioscience (Bristol, UK). U0126 was used at 20 μM. Controls were treated with DMSO.

### Shp2 siRNA and plasmid and transient transfection

Shp2-control and specific siRNAs were from Dharmacon RNA Technologies (Lafayette, CO); the oligonucleotide sequences were the following: 5′-GAACAUCACGGCAAUUAAUU-3′ and 5′-GAACACUGGUGAUUACUAUUU-3′. Human wild-type Shp2 (Shp2^WT^) cDNA was a generous gift from Prof. Benjamin Peng (Hong Kong University of Science and Technology, Hong Kong SAR, China); the cDNA was cloned into pSP64R1 vector. The siRNAs and the Shp2^WT^ plasmid were transfected into cells by using Lipofectamine 2000 (Invitrogen Life Technologies, Carlsbad, CA) according to the protocol recommended by the manufacturer. Immunoblotting was performed to assess Shp2 silencing or overexpression at 72 h after transfection.

### Cell-proliferation assay

Cell proliferation was measured by performing the CCK8 assay according to the manufacturer’s specifications (DOJINDO, Kumamoto-ken, Japan). Briefly, cells seeded in 96-well plates were treated with up to 10 μM gefitinib for 3 days. Subsequently, 110 μL of fresh medium containing 10 μL of CCK8 reagent was added to the wells, and the plates were incubated for 2 h at 37°C. The culture plates were then placed on a microplate reader, and the optical density (OD) was measured at 450 nm. Wells containing only medium were used for background correction. Each experiment was performed at least thrice.

### Immunoblotting

Cells exposed to different treatments were lysed on ice for 1 h in RIPA buffer, and the extracts were used in western blotting analyses. Proteins were resolved using SDS-PAGE and transferred to PVDF membranes (NEN, Boston, MA), which were blocked in TBS containing 5% skim milk (Sigma, St. Louis, MO) and then incubated (overnight, 4°C) with mouse monoclonal antibodies against Shp2 (1:1000; 610622, BD Transduction Laboratories, San Jose, CA), c-Myc (1:1000; sc-40, Santa Cruz Biotechnology), E-cadherin (1:500; 610252, BD Transduction Laboratories), or GAPDH (1:1000; Zhongshan Ltd., Beijing, China); or rabbit polyclonal antibodies against fibronectin (1:250; ab2413, Abcam, Cambridge, UK) or ERK or p-ERK (both 1:1000; Cell Signaling Technology, Danvers, MA). Immunoreactive bands were detected by incubating membranes with HRP-conjugated secondary antibodies (1 h, room temperature) and then with enhanced chemiluminescence substrate.

### Cell-migration assays

Firstly, cells were starved for 24h in RPMI medium alone. Wound-healing assay: Confluent cell monolayers in 6-well plates were scratched with a 200-μL pipet tip and then incubated for 24 h in RPMI medium alone. Scratch areas were quantified using Image Pro Plus. Where U0126 was used, cells were plated at sub-confluence and treated on Day 3 with medium containing U0126. Transwell-migration assay: After different treatments, 10^5^ cells were resuspended in RPMI medium alone and added to Transwell membranes (diameter: 6.5 mm; pore size: 8 μm; Corning, Corning, NY), which were placed in 24-well-plate wells containing 700 μL of RPMI medium supplemented with 10% FBS. The chambers were incubated for 24 h at 37°C. The cells that migrated to the lower chamber were fixed in 4% paraformaldehyde for 30 min and stained with crystal violet. The invasion-assay results were quantified by counting the cells on the lower surface of the filters by using Image Pro Plus.

### Statistical analysis

All data were analyzed using Student’s *t* test and are presented as means ± SD. Gefitinib IC50 values were calculated by fitting a 3-parameter logistic function to normalized data. Differences were considered statistically significant at *P* < 0.05. All statistical analyses were performed using SPSS version 16.0 software.

## Abbreviations

NSCLC: non-small cell lung cancer
TKI: tyrosine kinase inhibitor
EGFR: epidermal growth factor receptor
PTK: protein tyrosine kinase
PTP: protein tyrosine phosphatase
Shp2: src homology 2 domain-containing tyrosine phosphatase 2
EMT: epithelial-to-mesenchymal transition

## References

[1] Torre LA, Bray F, Siegel RL, Ferlay J, Lortet-Tieulent J, Jemal A (2015). Global cancer statistics, 2012. CA Cancer J Clin.

[2] D'Amico TA (2004). Angiogenesis in non-small cell lung cancer. Semin Thorac Cardiovasc Surg.

[3] Green MR (2004). Targeting targeted therapy. N Engl J Med.

[4] Jiang H (2009). Overview of gefitinib in non-small cell lung cancer: an Asian perspective. Jpn J Clin Oncol.

[5] Buonato JM, Lazzara MJ (2014). ERK1/2 blockade prevents epithelial-mesenchymal transition in lung cancer cells and promotes their sensitivity to EGFR inhibition. Cancer Res.

[6] Hunter T (2009). Tyrosine phosphorylation: thirty years and counting. Curr Opin Cell Biol.

[7] Neel BG, Gu H, Pao L (2003). The 'Shp'ing news: SH2 domain-containing tyrosine phosphatases in cell signaling. Trends Biochem Sci.

[8] Bentires-Alj M, Paez JG, David FS, Keilhack H, Halmos B, Naoki K, Maris JM, Richardson A, Bardelli A, Sugarbaker DJ, Richards WG, Du J, Girard L (2004). Activating mutations of the noonan syndrome-associated SHP2/PTPN11 gene in human solid tumors and adult acute myelogenous leukemia. Cancer Res.

[9] Wang FM, Liu HQ, Liu SR, Tang SP, Yang L, Feng GS (2005). SHP-2 promoting migration and metastasis of MCF-7 with loss of E-cadherin, dephosphorylation of FAK and secretion of MMP-9 induced by IL-1beta *in vivo* and *in vitro*. Breast Cancer Res Treat.

[10] Furcht CM, Munoz Rojas AR, Nihalani D, Lazzara MJ (2013). Diminished functional role and altered localization of SHP2 in non-small cell lung cancer cells with EGFR-activating mutations. Oncogene.

[11] Gagne-Sansfacon J, Coulombe G, Langlois MJ, Langlois A, Paquet M, Carrier J, Feng GS, Qu CK, Rivard N (2016). SHP-2 phosphatase contributes to KRAS-driven intestinal oncogenesis but prevents colitis-associated cancer development. Oncotarget.

[12] Chan G, Kalaitzidis D, Neel BG (2008). The tyrosine phosphatase Shp2 (PTPN11) in cancer. Cancer Metastasis Rev.

[13] Grosskopf S, Eckert C, Arkona C, Radetzki S, Bohm K, Heinemann U, Wolber G, von Kries JP, Birchmeier W, Rademann J (2015). Selective inhibitors of the protein tyrosine phosphatase SHP2 block cellular motility and growth of cancer cells *in vitro* and *in vivo*. ChemMedChem.

[14] Leibowitz MS, Srivastava RM, Andrade Filho PA, Egloff AM, Wang L, Seethala RR, Ferrone S, Ferris RL (2013). SHP2 is overexpressed and inhibits pSTAT1-mediated APM component expression, T-cell attracting chemokine secretion, and CTL recognition in head and neck cancer cells. Clin Cancer Res.

[15] Wang HC, Chiang WF, Huang HH, Shen YY, Chiang HC (2014). Src-homology 2 domain-containing tyrosine phosphatase 2 promotes oral cancer invasion and metastasis. BMC Cancer.

[16] Aceto N, Sausgruber N, Brinkhaus H, Gaidatzis D, Martiny-Baron G, Mazzarol G, Confalonieri S, Quarto M, Hu G, Balwierz PJ, Pachkov M, Elledge SJ, van Nimwegen E (2012). Tyrosine phosphatase SHP2 promotes breast cancer progression and maintains tumor-initiating cells via activation of key transcription factors and a positive feedback signaling loop. Nat Med.

[17] Breitkopf SB, Yang X, Begley MJ, Kulkarni M, Chiu YH, Turke AB, Lauriol J, Yuan M, Qi J, Engelman JA, Hong P, Kontaridis MI, Cantley LC (2016). A Cross-species study of PI3K protein-protein interactions reveals the direct interaction of P85 and SHP2. Sci Rep.

[18] Srikar R, Suresh D, Zambre A, Taylor K, Chapman S, Leevy M, Upendran A, Kannan R (2016). Targeted nanoconjugate co-delivering siRNA and tyrosine kinase inhibitor to KRAS mutant NSCLC dissociates GAB1-SHP2 post oncogene knockdown. Sci Rep.

[19] Xu J, Zeng LF, Shen W, Turchi JJ, Zhang ZY (2013). Targeting SHP2 for EGFR inhibitor resistant non-small cell lung carcinoma. Biochem Biophys Res Commun.

[20] Madhavan R, Zhao XT, Reynolds AB, Peng HB (2006). Involvement of p120 catenin in myopodial assembly and nerve-muscle synapse formation. J Neurobiol.

[21] Xiao X, Zhao XT, Xu LC, Yue LP, Liu FY, Cai J, Liao FF, Kong JG, Xing GG, Yi M, Wan Y (2015). Shp-1 dephosphorylates TRPV1 in dorsal root ganglion neurons and alleviates CFA-induced inflammatory pain in rats. Pain.

[22] Zhao XT, Qian YK, Chan AW, Madhavan R, Peng HB (2007). Regulation of ACh receptor clustering by the tyrosine phosphatase Shp2. Dev Neurobiol.

[23] Seo AN, Yang JM, Kim H, Jheon S, Kim K, Lee CT, Jin Y, Yun S, Chung JH, Paik JH (2014). Clinicopathologic and prognostic significance of c-MYC copy number gain in lung adenocarcinomas. Br J Cancer.

[24] Schneeberger VE, Luetteke N, Ren Y, Berns H, Chen L, Foroutan P, Martinez GV, Haura EB, Chen J, Coppola D, Wu J (2014). SHP2E76K mutant promotes lung tumorigenesis in transgenic mice. Carcinogenesis.

[25] Li Z, Owonikoko TK, Sun SY, Ramalingam SS, Doetsch PW, Xiao ZQ, Khuri FR, Curran WJ, Deng X (2012). c-Myc suppression of DNA double-strand break repair. Neoplasia.

[26] Rapp UR, Korn C, Ceteci F, Karreman C, Luetkenhaus K, Serafin V, Zanucco E, Castro I, Potapenko T (2009). MYC is a metastasis gene for non-small-cell lung cancer. PLoS One.

[27] Soucek L, Whitfield JR, Sodir NM, Masso-Valles D, Serrano E, Karnezis AN, Swigart LB, Evan GI (2013). Inhibition of Myc family proteins eradicates KRas-driven lung cancer in mice. Genes Dev.

[28] Paez JG, Janne PA, Lee JC, Tracy S, Greulich H, Gabriel S, Herman P, Kaye FJ, Lindeman N, Boggon TJ, Naoki K, Sasaki H, Fujii Y (2004). EGFR mutations in lung cancer: correlation with clinical response to gefitinib therapy. Science.

[29] Nurwidya F, Takahashi F, Murakami A, Kobayashi I, Kato M, Shukuya T, Tajima K, Shimada N, Takahashi K (2014). Acquired resistance of non-small cell lung cancer to epidermal growth factor receptor tyrosine kinase inhibitors. Respir Investig.

[30] Uramoto H, Sugio K, Oyama T, Sugaya M, Hanagiri T, Yasumoto K (2006). Resistance to gefitinib. Int J Clin Oncol.

[31] Yun CH, Mengwasser KE, Toms AV, Woo MS, Greulich H, Wong KK, Meyerson M, Eck MJ (2008). The T790M mutation in EGFR kinase causes drug resistance by increasing the affinity for ATP. Proc Natl Acad Sci U S A.

[32] Chen B, Xiao F, Li B, Xie B, Zhou J, Zheng J, Zhang W (2013). The role of epithelial-mesenchymal transition and IGF-1R expression in prediction of gefitinib activity as the second-line treatment for advanced nonsmall-cell lung cancer. Cancer Invest.

[33] Yoo SB, Kim YJ, Kim H, Jin Y, Sun PL, Jheon S, Lee JS, Chung JH (2013). Alteration of the E-cadherin/beta-catenin complex predicts poor response to epidermal growth factor receptor-tyrosine kinase inhibitor (EGFR-TKI) treatment. Ann Surg Oncol.

[34] Okumura T, Asamura H, Suzuki K, Kondo H, Tsuchiya R (2001). Intrapulmonary metastasis of non-small cell lung cancer: a prognostic assessment. J Thorac Cardiovasc Surg.

[35] Wang Y, Sheng Q, Spillman MA, Behbakht K, Gu H (2012). Gab2 regulates the migratory behaviors and E-cadherin expression via activation of the PI3K pathway in ovarian cancer cells. Oncogene.

[36] Tang C, Luo D, Yang H, Wang Q, Zhang R, Liu G, Zhou X (2013). Expression of SHP2 and related markers in non-small cell lung cancer: a tissue microarray study of 80 cases. Appl Immunohistochem Mol Morphol.

[37] Grande M, Franzen A, Karlsson JO, Ericson LE, Heldin NE, Nilsson M (2002). Transforming growth factor-beta and epidermal growth factor synergistically stimulate epithelial to mesenchymal transition (EMT) through a MEK-dependent mechanism in primary cultured pig thyrocytes. J Cell Sci.

[38] Shin S, Dimitri CA, Yoon SO, Dowdle W, Blenis J (2010). ERK2 but not ERK1 induces epithelial-to-mesenchymal transformation via DEF motif-dependent signaling events. Mol Cell.

